# Electrochemical Properties of Carbon Aerogel Electrodes: Dependence on Synthesis Temperature

**DOI:** 10.3390/molecules24213847

**Published:** 2019-10-25

**Authors:** Alena N. Malkova, Nataliya A. Sipyagina, Inna O. Gozhikova, Yury A. Dobrovolsky, Dmitry V. Konev, Alexander E. Baranchikov, Olga S. Ivanova, Alexander E. Ukshe, Sergey A. Lermontov

**Affiliations:** 1 Institute of Physiologically Active Compounds of the Russian Academy of Sciences, 1 Severnij pr., Chernogolovka 142432, Russia; amalkova81@gmail.com (A.N.M.); dolmatin_89@mail.ru (N.A.S.); innagozhik@gmail.com (I.O.G.); 2 Institute of Problems of Chemical Physics of the Russian Academy of Sciences, 1 Acad. Semenov av., 1, Chernogolovka 142432, Russia; dobr62@mail.ru (Y.A.D.); dkfrvzh@gmail.com (D.V.K.); ukshe@mail.ru (A.E.U.); 3 Kurnakov Institute of General and Inorganic Chemistry of the Russian Academy of Sciences, 31 Leninsky av., Moscow 119991, Russia; a.baranchikov@yandex.ru (A.E.B.); runetta05@mail.ru (O.S.I.)

**Keywords:** carbon aerogel, supercapacitor, electrode material, electron conductivity, specific capacitance

## Abstract

A series of carbon aerogels (C-AGs) were prepared by the pyrolysis of resorcinol-formaldehyde aerogels at 700–1100 °C as potential supercapacitor electrodes, and their texture and electrochemical properties were determined. The specific surface area of all C-AGs was in the range of 700–760 m^2^/g, their electron conductivity increased linearly from 0.4 to 4.46 S/cm with an increase of the pyrolysis temperature. The specific capacitance of electrode material based on C-AGs reached 100 F/g in sulfuric acid and could be realized at a 2 A/g charge-discharge current, which makes it possible to use carbon aerogels as electrode materials.

## 1. Introduction

In recent years, new carbon materials have attracted increased attention due to their excellent properties. One of the promising materials is carbon aerogel (C-AGs), which is a 3D-network of nanoscale particles. Such materials are usually synthesized by the pyrolysis of precursors, such as polymer organic aerogels [1]. The most common precursor is resorcinol-formaldehyde resin (RF-AG), which is easily obtained by polycondensation of a mixture of resorcinol and formaldehyde under alkali or acid catalysis conditions [2,3,4,5,6,7,8].

Previously, C-AGs were used as electrode materials for the proton exchange membrane fuel cells (PEM FC) [7]. The use of C-AGs as an electrode material in electrochemical double-layer capacitors (supercapacitors) has been described in a number of works [9,10,11,12,13,14,15,16]. Thus, silicon carbide aerogels were treated with chlorine gas at 700–1000 °C to remove silicon and to form a highly porous carbon material with a specific surface area as high as 2700 m^2^/g. Electrochemical elements based on it had a specific capacitance up to 240 F/g and a current density of 20 A/g. The parameters are high, but the preparation procedure looks very dangerous and costly [9,10,11]. Similar material prepared by high-temperature chlorination of molybdenum carbide provided a ~125 F/g gravimetric capacitance value [15].

A number of commercial coals and carbon aerogel prepared at 800 °C and activated by KOH have been investigated recently as electrode materials [12]. Similar results were obtained for both activated carbon and aerogel: capacitance of approximately 30 F/g and equivalent series resistance (ESR) of nearly 5 Ω. Electrodes from carbon xerogels prepared at 1050 °C and based on enzymatically hydrolysed lignin were also investigated [13]. The specific capacitances up to 140 F/g with ESR of 0.5–0.8 Ohm in H_2_SO_4_ solution and up to 175 F/g and ESR of 20.7 Ohm in KOH were obtained. Mixed resorcinol-melamine-formaldehyde xerogels were pyrolysed at 700 °C and the N-doped C-xerogels obtained showed the largest capacitance of 139 F/g and an ESR of about 1.5 Ohm in a KOH solution [14]. Supercapasitor electrodes prepared from C-xerogels post-treated in air at 300 °C showed the specific capacitance of about 40 F/g [17].

For practical use in supercapacitors, carbon materials should have a large specific surface area and low electrical resistance of both the electrode material itself and the electrolyte in the pores as critically important characteristics. In fact, the energy capacity of supercapacitors is small compared to batteries, so they are used as energy sources with very high short-term power. So, a discharge of the double electric layer capacitance in a short time is required, determining the need for low “equivalent series resistance” (ESR) [18]. Therefore, in our study we have focused on determination and optimization of both parameters—specific capacity and ESR.

Earlier, the authors of [19] measured the capacities of carbon aerogel samples obtained by different synthesis methods, bearing in mind their use as supercapacitor electrodes. However, the electrical resistances of neither the material itself nor the entire cell were controlled.

Very often, comparison of the materials described in different papers is difficult, as the C-electrode preparation conditions (pyrolysis temperature, precursor composition, KOH activation) differ significantly. We decided to investigate the influence of only one parameter—the pyrolysis temperature of the initial resorcinol-formaldehyde aerogels obtained by the acid-catalyzed polycondensation reaction—on the texture and electrochemical properties of carbon aerogels and leaving other synthetic conditions unchanged.

## 2. Results and Discussion

**Figure 1:**
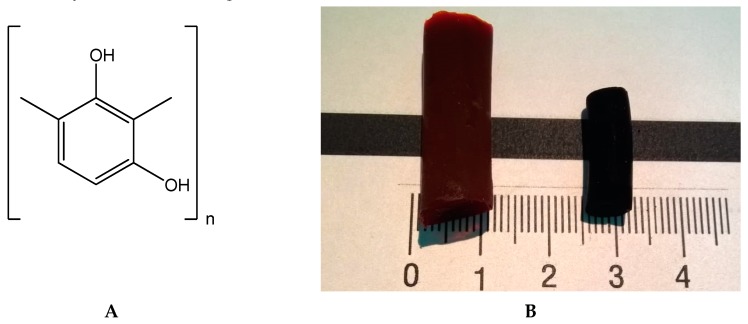
Resorcinol-formaldehyde (RF)-gel and aerogel chemical structure (**A**). RF-(left) and carbon aerogel (C-AGs) (right) (**B**). After cooling and washing with isopropanol all “wet” samples were dried in supercritical CO_2_ at 50 °C/150 atm. As a result dark-brown semitransparent RF-aerogels were obtained (Figure 1B, left). The pyrolysis of these samples was carried out in an atmosphere of nitrogen in the temperature range 700–1100 °C and resulted in C-AGs (Figure 1B, right). Hereafter the following notation is used: C-AGs prepared from RF-AGs at 700 °C are denoted RF-700, etc. C-AG linear size was approximately one and a half time smaller than that of parent RF-AGs (Figure 1B).

The molecular structure of the RF-gel and aerogel prepared by the literature method [8], can be described by the formula in Figure 1A.

### 2.1. Texture Properties

Some texture properties of RF-AGs and C-AGs are presented in Table 1.

It can be seen that a specific surface area of C-AGs is practically independent from annealing temperature and practically doubles the S_sp_ of initial RF-aerogel. The porosity of C-AGs was 91–93%.

**Figure 2:**
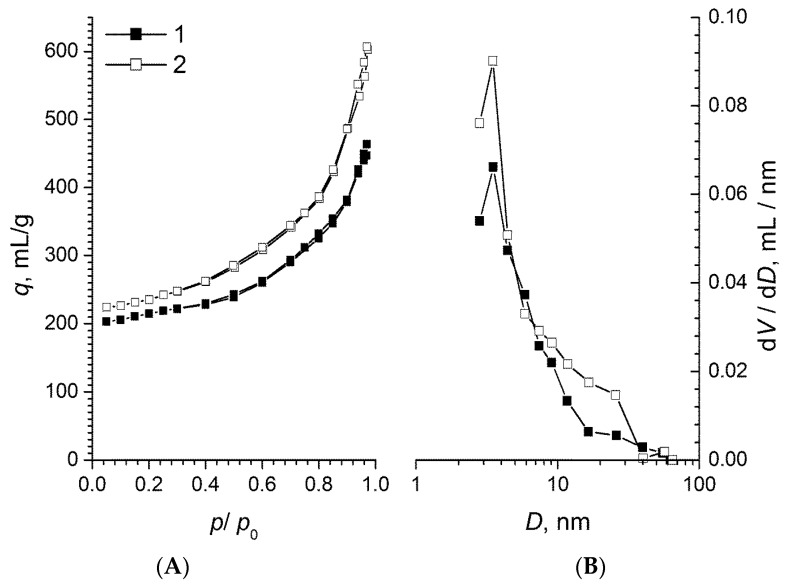
Full adsorption–desorption isotherms (**A**) and pore size distributions (**B**) for RF-700 (1) and RF-1100 (2) samples.

The full adsorption–desorption isotherms are presented in Figure 2.

Full adsorption-desorption isotherms for both samples correspond to type II according to IUPAC classification, which is typical for macroporous sorbents. Practically no hysteresis loops were present indicating a small amount of mesopores in the materials. The pore size distributions obtained from nitrogen desorption data indicate that the nanopore structure of both samples was practically identical, yet the sample annealed at 1100 °C possessed a higher mesoporosity in the 10–30 nm range.

### 2.2. Scanning Electron Microscopy

**Figure 3:**
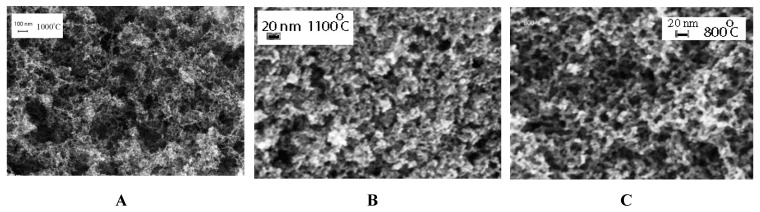
The porous microstructure of RF-1000 (**A**), RF-1100 (**B**) and RF-800 aerogels (**C**).

It can clearly be seen that C-AGs have a highly porous structure with 50–300 nm and 5–15 nm pores (Figure 3) and the change of the pyrolysis temperature did not noticeably affect the samples’ microstructure.

**Figure 4:**
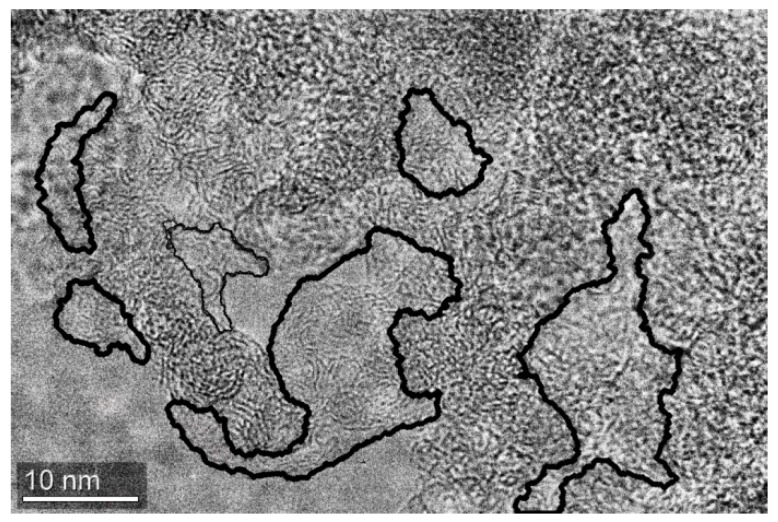
The TEM image of the RF-1100 sample. Graphene scales between carbon grains are visible in blue.

The TEM images of RF-1100 sample are presented at Figure 4. The boundaries of carbon grains are highlighted by a solarization filter revealing that the grain boundaries in these samples were wider, and grains begin to combine together (see Supplementary, Figure S4). It can be seen that the selected areas between grains were filled by some structures with a density lower than that in compact grains. 

This phenomenon looks like a peeling of graphene-like scales from the surface of carbon grains. The grains had no long-range order, as shown by the analysis of electron diffraction.

### 2.3. Electric and Electrochemical Parameters

#### 2.3.1. Electronic Conductivity

**Figure 5:**
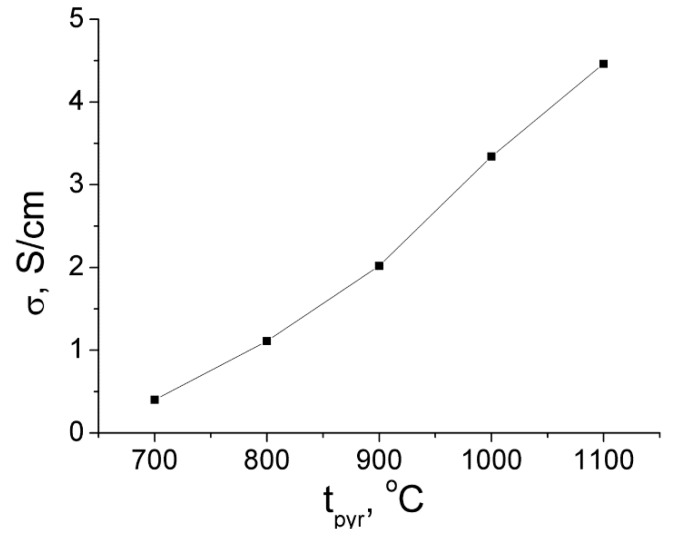
The dependence of specific electronic conductivity of carbon aerogels on pyrolysis temperature.

Electronic conductivity of the materials investigated grew linearly with an increase of the pyrolysis temperature. (Figure 5). The reason for this phenomenon is not yet clear, but it can be concluded that the resistance of the intergranular boundaries between the aerogel particles decreased with an increase in the pyrolysis temperature. The electrical conductivity of the AG samples was low (about 50 times less than the compact amorphous carbon—the specific electrical conductivity of the glassy carbon was about 200 S/cm).

#### 2.3.2. Characteristics of the Electrode Material from C-aerogels

**Figure 6:**
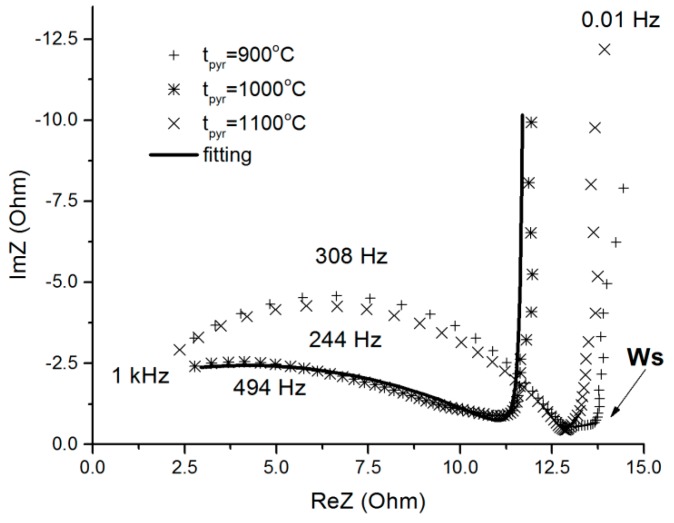
Spectrum of sample impedance (pyrolysis at 1000, 1100 and 900 °C) in the complex plane (hodograph). Z′ = RE (Z) is the real (active) component, Z″ = Im (Z) is the imaginary (reactive, capacitive) component.

The typical impedance spectra of RF-900, RF-1000 and RF-1100 samples are given in complex coordinates (Nyquist) in Figure 6. The spectra (hodographs) of RF-1000 and RF-1100 samples are similar, and can be divided into two regions. In the frequency domain, higher than 0.2 Hz there is a strong depressed arc (depressing angle is more than π/4), but lower than the 0.2 Hz domain there is a typical capacitive frequency response—an almost vertical straight line.

The results of adjustment of the equivalent circuit (Figure S1) elements are shown at Figure 6 as a solid line, adjustment parameters are presented in Table 2. In order to consider the process in the pores as diffusive, the value *p* = 0.5 (due to the square root of the time in the Fick equation) is necessary. In our case, *p* = 0.4, which is close to 0.5, the deviation can be explained by diffusion in a branched pore system.

Electrodes from RF-1000 and RF-1100 aerogels have close parameters, but RF-900 electrode demonstrates an additional relaxation component at 0.2 Hz (Ws arrow, Figure 6). We suppose that it is the resistivity of grain boundaries.

This fact required the insertion of the closed Warburg impedance (Ws) into the equivalent scheme (Figure S2) for the diffusion stage consideration.

The capacitance of 1.6 F per sample corresponds to the approx. 100 F/g specific capacitance of the material at the AC frequencies below 0.2 Hz, but the effective serial resistance (ESR) is rather high, up to 15 Ohm·cm for 3 mm electrode thickness.

#### 2.3.3. Direct Current Measurements

Capacitance parameter determination was performed using both the cyclic voltammetry (CVA) method and charge-discharge curve investigations.

- CVA measurements

The CVA measurements method was used for determination of two parameters: properties of the simple equivalent circuit (ESR) and the double layer capacitance (ESR includes both electrical resistance of carbon and ionic resistance of electrolyte in pores). ESR is the key characteristic for practical application. 

These parameters depend on the charge-discharge velocity that means-on the discharge current and determines the supercapacitor power. The reason is that small charge-discharge times, that is, high currents, do not include deep pores and do not allow using the entire electrode thickness effectively. This “long line” effect limits the power of the model ionistor.

**Figure 7:**
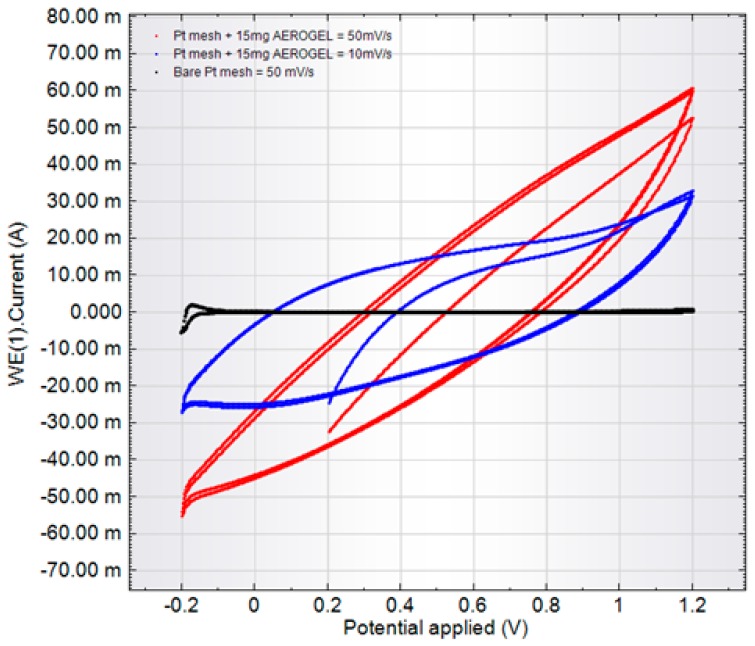
Voltammograms of samples of the experimental supercapacitor electrode from RF-1000 aerogel.

CVA curves for RF-1000 electrode are presented at Figure 7 (CVA curve for the empty adapter is presented as 3). The curves are of elliptical shape for the series RC-circuit. CVA curves for RF-900 and RF-1100 electrode see in Supplementary, Figures S4 and S5 correspondingly.

The capacitance value calculated for different scanning rates was as follows:

V = 50 mV/s, C = 0.35 F;

V = 10 mV/s, C = 1.07 F.

The estimation of series resistance for V = 10 mV/s gave the ESR value ~35 Ohm.

Supercapacitors are better characterized by CVA than by an impedance method, so the following parameters are more reliable from the point of view of technical application. Specific parameters and CVA-curve shapes were similar for all C-AG samples. The top efficient discharge rate does not exceed 10 mV/s, leading to a full discharge time of approximately 100 s. The measured specific capacitance was 70 F/g and ESR ~35 Ohm.

- Capacitance parameters determination by a current transient method (at constant potential)

**Figure 8:**
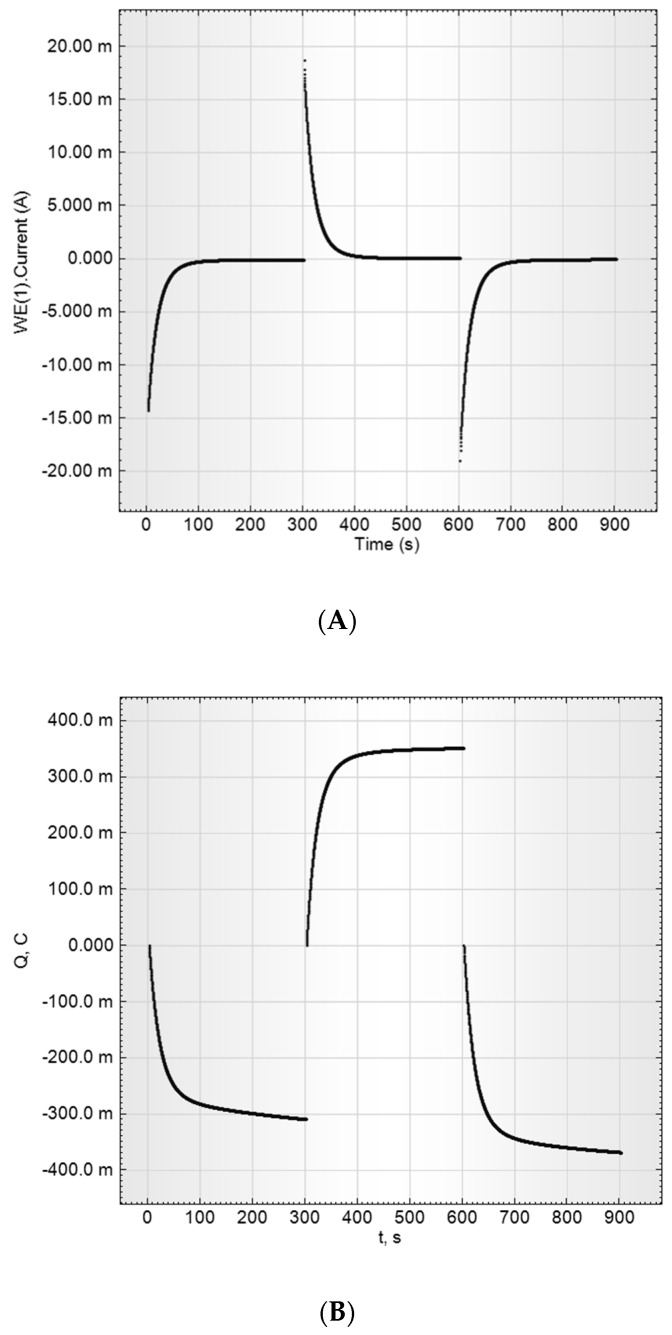
The charge-discharge curves of RF-1000 sample (15 mg) in a 2M aqueous sulfuric acid solution. (**A**) is the charging current vs. time I (t), (**B**) is the current integral, that is, the charge of the supercapacitor, Q (t).

Charge-discharge curves at constant potential (current transients) were also received for the estimation of aerogel materials as supercapacitors’ electrodes, namely ESR and capacitance. The potential impulse sequence was 200 mV→400 mV→200 mV with 300 s impulse duration. The current charge dependences on time are presented in Figure 8.

According to the curves presented, charge and discharge were symmetrical, 95% charge-discharge time was estimated as ~80 s, the efficiently used capacitance is 1.75 F and the specific capacitance is 117 F/g. The current drop at charge-discharge was not exponential, revealing strong diffusion limitations on the charge speed.

**Figure 9:**
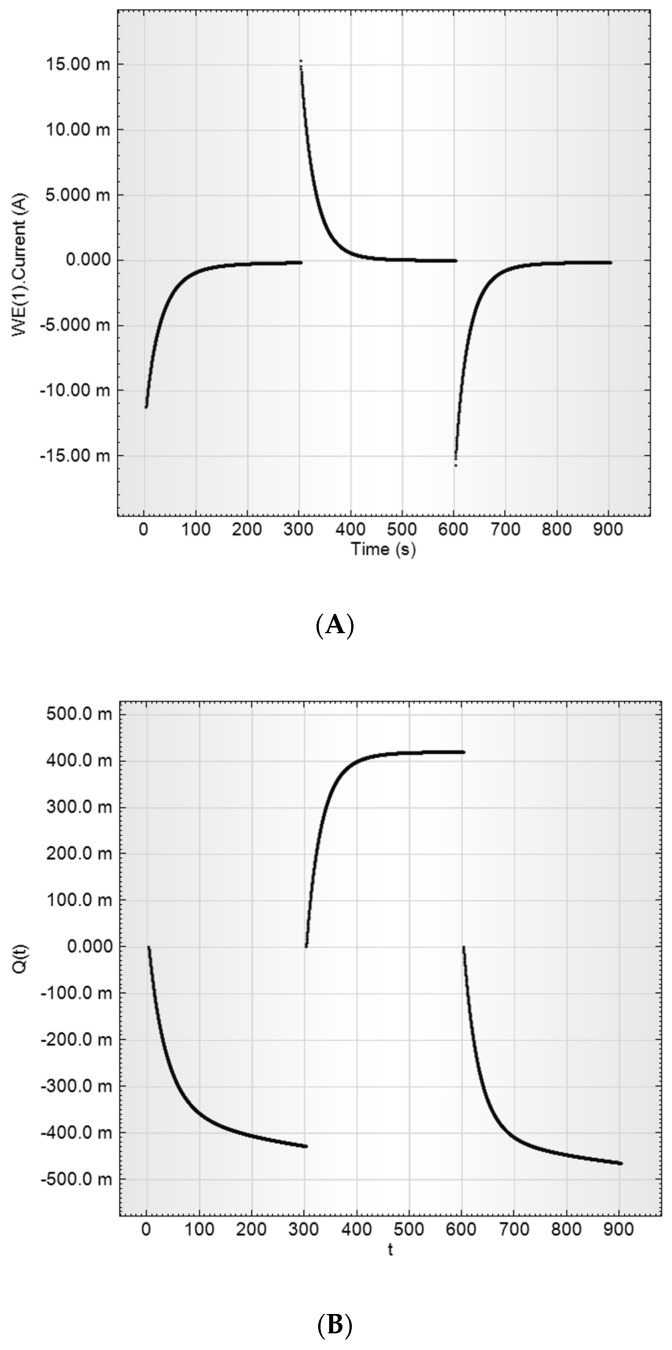
The charge-discharge curves of RF-900 sample (13.6 mg) in a 2M aqueous sulfuric acid solution. (**A**) is the charging current vs. time I (t). (**B**) is the current integral, that is, the charge of the supercapacitor, Q (t).

Specific parameters and curve shapes were identical for both RF-1000 and RF-1100 samples, but the charge-discharge curves for the RF-900 electrode look more prolonged (Figure 9).

The total charge time for the electrode increased but did not exceed 100 s. The charge-discharge curves for the RF-900 electrode were far from exponential, and the time constant in short times was small, τ ≈ 10 s. Specific capacitance was approximately 154 F/g, but the additional diffusion velocity was 1.5 times less than that of the RF-1000 and RF-1100 aerogels.

Summary of electrochemical parameters of all carbon aerogels is presented in Table 3.

The appearance of the specific capacitance maximum of the RF-900 sample (154 F/g) compared to RF-1000 and RF-1100 samples (117 F/g) is not yet clear, though the same regularity was observed earlier—the specific capacitance at 800 °C was much higher than that at 1000 and 1200 °C, while the electric conductivity increased with increase in temperature [17]. We suppose that the decrease of the specific capacitance with temperature increase was connected to the observed growth of graphene interlayers between grains (Figure 4). These interlayers block the grain’s surface and pores, making them electrochemically inaccessible, which leads to effective surface reduction. 

## 3. Materials and Methods 

### 3.1. Materials

Resorcinol (98%, Acros, NJ, USA), formaldehyde (37 wt.% solution in water, Acros, Morris, NJ, USA), isopropanol (99.5%+, Acros, Morris, NJ, USA), acetonitrile (99%+, Acros, Morris, NJ, USA), and HCl (37%, Acros, Morris, NJ, USA), were used as received.

### 3.2. Preparation of Sols

A standard resorcinol:formaldehyde 1:2 molar ratio was applied in all cases. First, 1.1 g (0.01 mol) of resorcinol was dissolved in 38 mL of acetonitrile in a plastic beaker, then 0.025 mL of HCl (38%) and 1.47 mL (0.02 mol) of formaldehyde solution were added, and the mixture was stirred for 10–20 min. 

### 3.3. Gelation of Sols

Sols (3–5 mL) were poured into polypropylene syringes of 3 or 5 mL volume. The gels were formed after 5–6 h at 20 °C. Upon formation, the gels were left to age at 50 °C for 7 days. The resultant aged gels were soaked in isopropanol for 24 h in order to exchange the pore liquid for the solvent. This procedure was repeated five times. Then the gels formed were placed into an apparatus for supercritical drying in CO_2_.

### 3.4. Supercritical Drying

Supercritical drying in CO_2_ was carried out in an installation composed of a high-pressure CO_2_ pump (SSI Supercritical 24, State College, PA, USA), a 50-mL steel reactor and Go Regulator BPR (Spartanburg, SC, USA) back pressure regulator. The isopropanol lyogel samples were washed with liquid CO_2_ for 2 h at 20 °C at a pressure of 15 MPa, then the temperature in the reactor was elevated to 50 °C, and the sample was washed with supercritical CO_2_ (15 MPa) for 2–2.5 h. Next, the pressure in the heated autoclave was gradually decreased to atmospheric pressure; the autoclave was cooled to ambient temperature and opened.

### 3.5. Pyrolysis Procedure

Cylindrical C-AG samples were prepared by pyrolysis of RF-AGs at 700–1100 °C in an inert atmosphere (N2) in a quartz flow reactor. The temperature was raised to a desired value over 1.5–2 h, the AG sample was kept for 4 h, and then the reactor was cooled to ambient temperature.

### 3.6. Characterization of Aerogels

The specific surface area of the aerogels was determined by low-temperature nitrogen adsorption measurements with a Katakon ATX-06 analyzer (Katakon, Novosibirsk, Russia), using an 8-point BET method. Experimental values were plotted against P/P_0_ according to the BET equation; the correlation coefficient of the linear regression was not less than 0.9975. Pore size distributions were deduced using the BJH method from full nitrogen adsorption-desorption isotherms (28 points).

The bulk densities of the samples were calculated using their mass to volume ratio. The reproducibility of different parallel synthesis was 7–11%.

The microstructure of the samples was studied using a field emission scanning electron microscope Karl Zeiss LEO SUPRA 25 (Carl Zeiss AG, Oberkochen, Germany). The transmission electron microscope JEM-2100 (JEOL) (JEOL, Tokyo, Japan) at different acceleration voltage (magnifications up to 400,000) was also used. The last device also gives the possibility to identify crystallites using electron diffraction analysis.

X-ray diffraction (XRD) analysis was carried out on a DRON diffractometer (Cu-Kα radiation) (Burevestnik, Saint Petersburg, Russia) in a 10–80° 2θ range at goniometer rotation speed of 5° 2θ/min.

The surface optical IR-band investigation was carried out by the method of attenuated total reflection, ATR-FTIR, on a vacuum Fourier spectrometer, Vertex-70V (Bruker, Stuttgart, Germany) in a 4000–50 cm^−1^ region.

Testing the electron conductivity of the aerogels was carried out using a four-point probe method (direct current) in a custom-made cell (see Supplementary).

The measurements of electrochemical characteristics of electrodes prepared from the aerogels investigated were carried out by three methods: cyclic voltammetry (CVA), charge-discharge (current transients) measurements and impedance spectroscopy (see Supplementary). The electrodes were prepared using the following procedure. The aerogel samples after pyrolysis were neatly cut into cylindrical pieces (Ø5 × 5 mm). Each cylindrical sample was wrapped in a platinum grid cylinder playing a role of an electrode (the platinum grid was tested as an “empty” electrode in all experiments). A sample in a platinum grid was placed into a 2 M H_2_SO_4_ aqueous solution and was impregnated by this solution via repeated careful evacuation of the flask.

## 4. Conclusions

The specific capacitance of the electrode material based on carbon aerogel in sulfuric acid is approximately 100 F/g, and can be realized at a sufficiently low charge-discharge rate. It follows from the literature [20] that carbon materials in sulfuric acid have a capacitance of about 0.1 F/m^2^, which corresponds to our results (0.132 F/m^2^). This confirms a conclusion that we observe a true double layer capacitance, rather than the pseudo capacitance due to chemisorption and/or proton injection.

Close specific capacitance values were obtained in [19], but our work includes ESR measurements, allowing the possibility of estimating the use of carbon aerogels in the form of massive material as supercapacitors’ electrodes.

Thus, the main process responsible for the slow charge is the diffusion in pores. This fact gives the possibility to expect that it will be possible to increase charge-discharge rates and significantly reduce the equivalent series resistance of the supercapacitor by optimization of the pore size, as well as by the use of the material in the form of a thin film.

The authors also believe that the observed dependence of the electrical and electrochemical parameters on the aerogel pyrolysis temperature was associated with poor grain boundary contacts in aerogels obtained at temperatures below 1000 °C. Thus, in spite of the higher specific capacitance of low-temperature samples, aerogels, obtained by high-temperature pyrolysis, should be used to develop supercapacitors, since the best contact between aerogel nanoparticles greatly improves the important temporal and power parameters of the electrode.

## Figures and Tables

**Table 1:** Specific surface area (m^2^/g) and bulk density (g/cm^3^) of RF-aerogels.

Series	S_sp,_ m^2^/g	Density, g/cm^3^
RF	430 ± 40	0.14 ± 0.02
RF-700	700 ± 70	0.16 ± 0.01
RF-800	730 ± 80	0.18 ± 0.02
RF-900	760 ± 80	0.15 ± 0.01
RF-1000	760 ± 80	0.14 ± 0.01
RF-1100	740 ± 80	0.15 ± 0.01

**Table 2:** Adjusted equivalent scheme parameters for samples, prepared at different temperature *.

	RF-900	RF-1000	RF-1100
R1, Ohm	11.3 ± 0.2	15.29 ± 0,04	13.47 ± 0.04
T	0.000144 ± 0.000004	0.00287 ± 0.00008	0.00038 ± 0.00001
W	6900 ± 200	350 ±10	2631 ± 70
p	0.845 ± 0.006	0.393 ± 0.003	0.716 ± 0.0004
C1, F	2.07 ± 0.01	1.59 ± 0.02	1.25 ± 0.0015

* The parameters of the diffusion element CPE in the table are: W (Warburg constant) is W=RTx2c0iωDp.

**Table 3:** The basic electrochemical properties of samples, prepared with different temperatures.

Method *		RF-900	RF-1000	RF-1100
impedance	R, Ohm	11.3 ± 0.2	15.29 ± 0.04	13.47 ± 0.04
transient	ESR, Ohm	26	35	32
impedance	W(diffusion) Ohm/Hz-^p^	6900 ± 200	350 ± 10	2631 ± 70
impedance	p	0.845 ± 0.006	0.393 ± 0.003	0.716 ± 0.0004
impedance	C, F	2.07 ± 0.01	1.59 ± 0.02	1.25 ± 0.0015
CVA	C, F	1.39	1.07	1.14
transient	C, F	2.1	1.75	1.35
impedance	C(sp), F/g	152	106	107
CVA	C(sp), F/g	102	71	97
transient	C(sp), F/g	154	117	115
transient	t_discharge_95%, sec	>100	80	50

* Capacitance by impedance method is determined at 10–100 sec; by cyclic voltammetry (CVA)—at 70 sec, and by transients method—at ~100 sec.

## Supplementary Materials

The following are available online. 1. The electrical conductivity measurements, Figure S1. 1—sample, 2—wires potential electrodes, 3—wires tensioning system, 4—current electrodes, bronchial clamp, 2. The electrochemical characteristics measurements, Figure S2. Equivalent circuit for the impedance spectra of an aerogel electrode prepared by pyrolysis at 1000 and 1100 °C, Figure S3. Equivalent circuit for the impedance spectra of an aerogel electrode prepared by pyrolysis at 900 °C, Figure S4 Voltammograms of samples of the experimental supercapacitor electrode from **RF-900** aerogel, Figure S5. Voltammograms of samples of the experimental supercapacitor electrode from **RF-1100** aerogel, Figure S6. The TEM images of samples, prepared at different pyrolysis temperatures. Samples were grounded and placed on amorphous carbon substrate, Figure S7. Powder X-ray diffraction patterns of **RF-800** (down) and **RF-1100** aerogels (up), Figure S8. The ATR-FTIR spectrum of **RF-1100** aerogel surface.
