# Supplementary material for: Electrochemical Properties of Carbon Aerogel Electrodes: Dependence on Synthesis Temperature
Source: Molecules. 2019 Oct 25;24(21):3847. doi: 10.3390/molecules24213847 (PMC6864835; doi:10.3390/molecules24213847)
Supplement: Supplementary file 1 [file molecules-24-03847-s001.pdf]

Supplementary

1. The electrical conductivity measurements

The electron conductivity of aerogels was carried out by a four-point probe method (direct current) in a custom-made cell (Figure S1). A cylindrical sample was clamped between two brass contacts of a 4-contact cell; the conductive carbon glue with a specific resistance of 3 Ohm*cm was used. The thin tinned copper wires were used as potential electrodes. The distance between wires (1 – 2 mm) was measured with an accuracy of 0.1 mm in each case. The measurements were carried out at ambient temperature under measuring current of 1 mA.

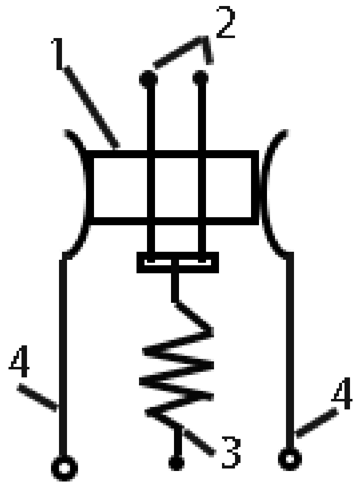

Figure S1. 1—sample, 2—wires potential electrodes, 3—wires tensioning system, 4—current electrodes, bronchial clamp.

2. The electrochemical characteristics measurements

A cylindrical aerogel sample (Ø5 × 5 mm) was wrapped in a platinum grid cylinder playing a role of an electrode. The platinum grid was tested as an "empty" electrode in all experiments. A sample in a platinum grid was placed into a 2M H_2SO_4 aqueous solution and was impregnated with this solution by repeated careful evacuation of the flask.

The measurements were carried out in an electrochemical liquid cell with platinum counter electrode (1 cm^2 surface) and reference electrode (the platinized platinum wire). All measurements were carried out in a degased electrolyte on an Autolab 301N (Metrohm Autolab) potentiostat with frequency response analysis unit for impedance measurements. All measurements were done at room temperature (28 °C).

Direct current data were received using Cyclic Voltammetry (CVA) and charge-discharge (current transients) measurements. CVA was measured at two different potential sweep speeds: 50 mV/s and 10 mV/s, in a potential range −0.2–1.2 V referring to the standard hydrogen electrode (SHE).

The negative potential was limited by hydrogen evolution as the result of water electrolysis. The initial potential (open-circuit potential, OCP) of all samples in experimental cell was about 200 mV referring to SHE.

The impedance of samples was measured in potentiostatic mode under external 200 mV potential referring to SHE (zero to OCP) and at 5mV amplitude of a signal in the frequency range 0.01 Hz–1 kHz.

The analysis of impedance spectra of aerogel samples, prepared at high pyrolysis temperature (1000 and 1100 °C) was performed with a use of the equivalent circuit Figure S2.

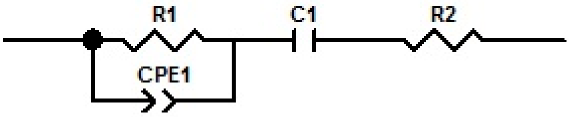

Figure S2. Equivalent circuit for the impedance spectra of an aerogel electrode prepared by pyrolysis at 1000 and 1100°C.

For an adjustment of impedance spectra of aerogel electrode, prepared at 900 °C, we used the equivalent circuit (Figure S3), which had an additional diffusion element, the limited closed generalized Warburg element W1. This element describes the diffusion relaxation impedance in thin layers and has the frequency dependence

$$Z = R\tanh(\frac{j\omega}{W})^p \Big/ (\frac{j\omega}{W})^p$$

where R is the closing resistance, equal to 3 ± 0.5 Ohm, and Warburg coefficient 0.5 ± 0.1 Ohm/Hz^p, where exponent p = 0.18 ± 0.03. As one can see, the exponent is very small so this limited Warburg element in fact describes the additional resistance with a small diffusion additive.

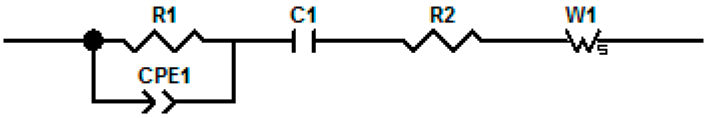

Figure S3. Equivalent circuit for the impedance spectra of an aerogel electrode prepared by pyrolysis at 900 °C.

The capacitance value calculated for different scanning rates is as follows:
V = 50 mV/s, C = 0.58 F;
V = 10 mV/s, C = 1.39 F.
The estimation of series resistance for V = 10 mV/s gives the ESR value ~26 Ohm.

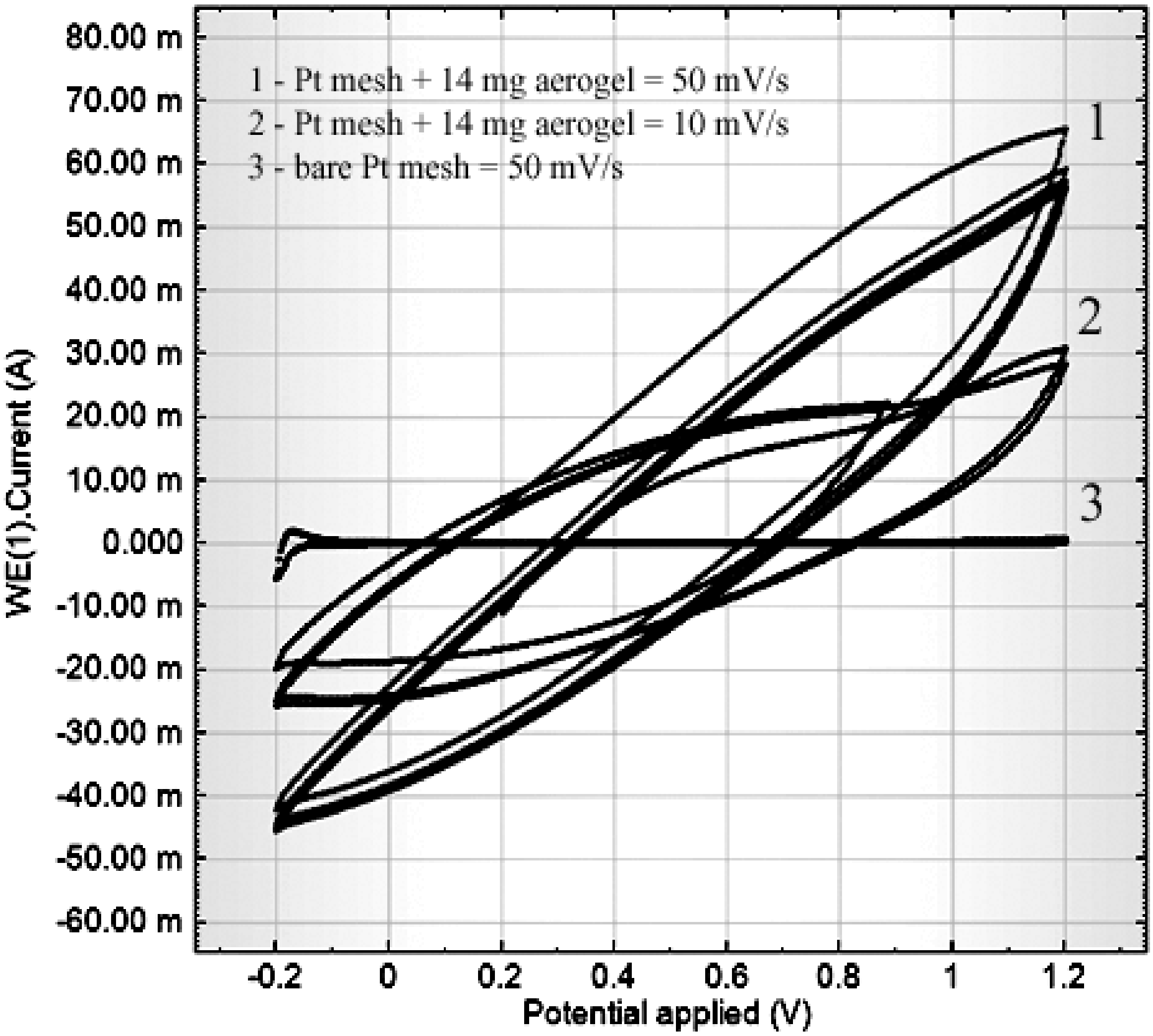

Figure S4. Voltammograms of samples of the experimental supercapacitor electrode from **RF-900** aerogel.

The capacitance value calculated for different scanning rates is as follows:
V = 50 mV/s, C = 0.62 F;
V = 10 mV/s, C = 1.14 F.
The estimation of series resistance for V = 10 mV/s gives the ESR value ~ 32 Ohm.

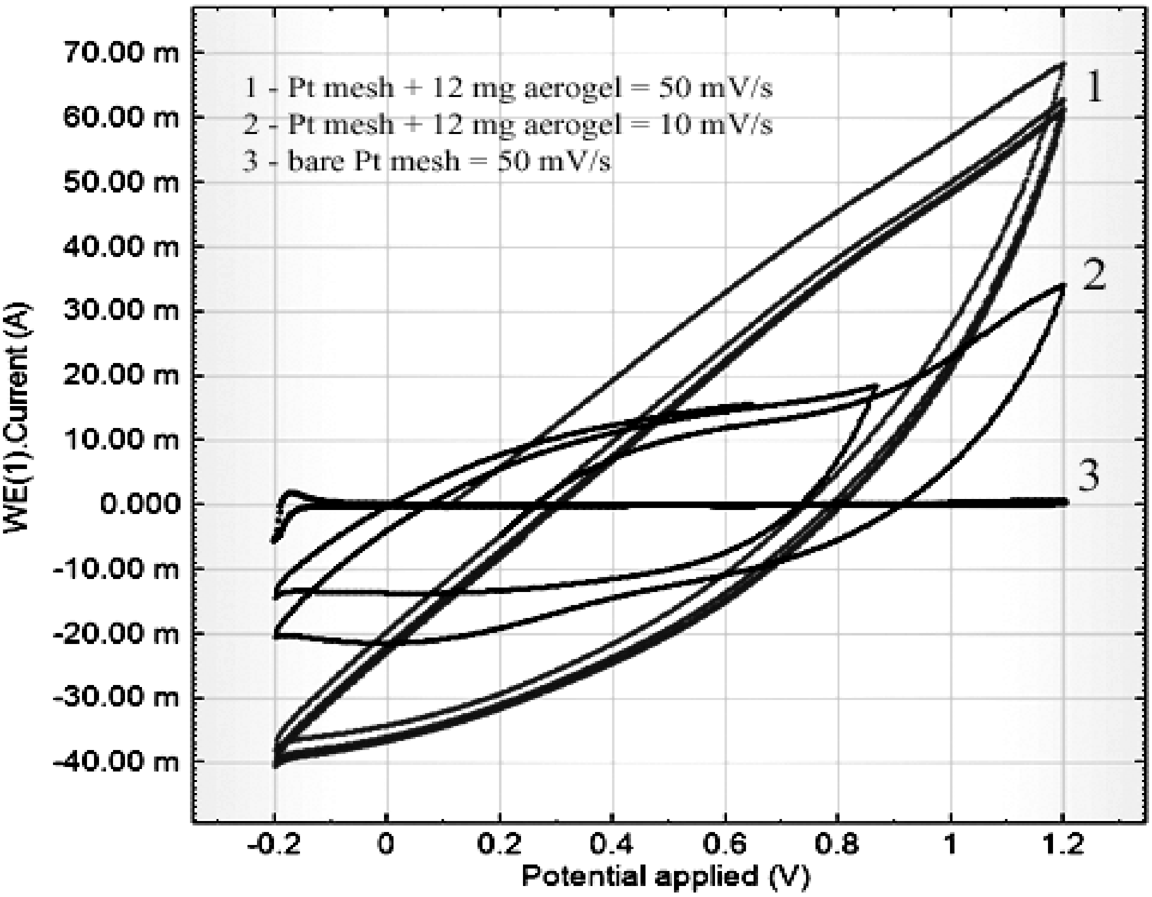

Figure S5. Voltammograms of samples of the experimental supercapacitor electrode from **RF-1100** aerogel.

3. The transmission electron microscope images of aerogels prepared at different temperatures

The TEM images are presented at Figure S6. One can see that there are no visible difference between samples prepared at 900 and 1000 °C.

	900 °C	1000 °C
	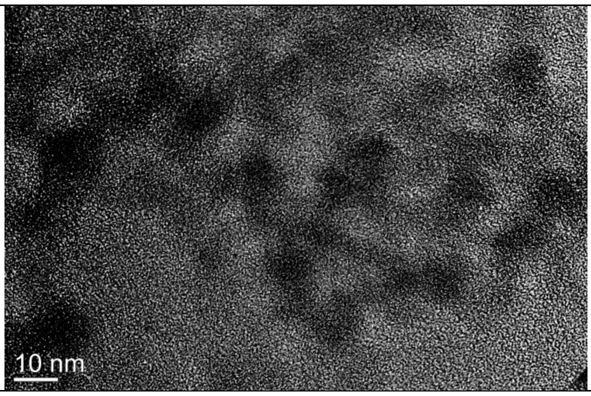	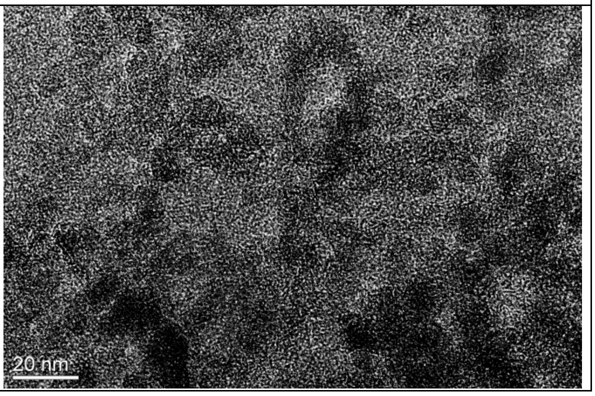

	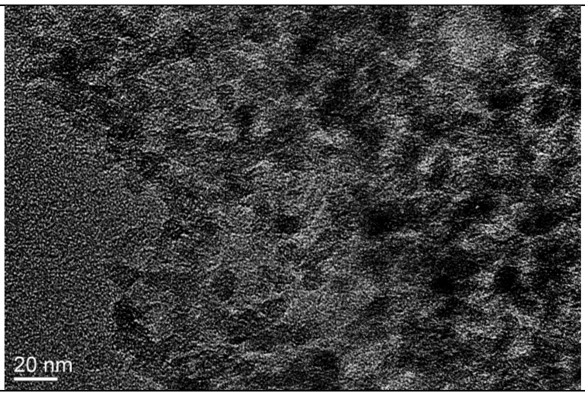	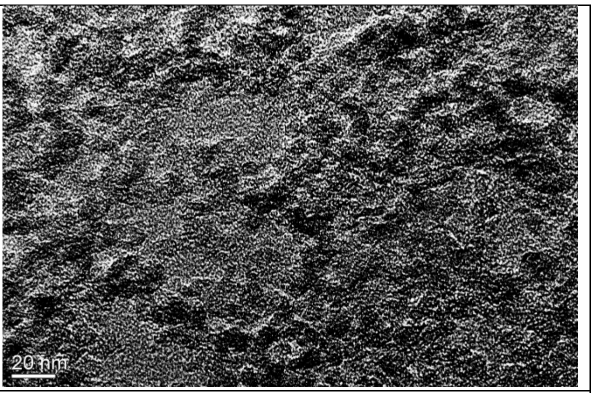
	1100 °C	

Figure S6. The TEM images of samples, prepared at different pyrolysis temperatures. Samples were grounded and placed on amorphous carbon substrate.

4. X-Ray diffraction investigation of material

Powder X-ray diffraction patterns of aerogels, prepared at different pyrolysis temperatures are presented at Figure S7. One can see, that the residual crystallinity is observed for the sample, prepared at 800 °C, but the **RF-1100** sample is absolutely amorphous. The observed peaks for **RF-800** do not correspond to the graphite peaks.

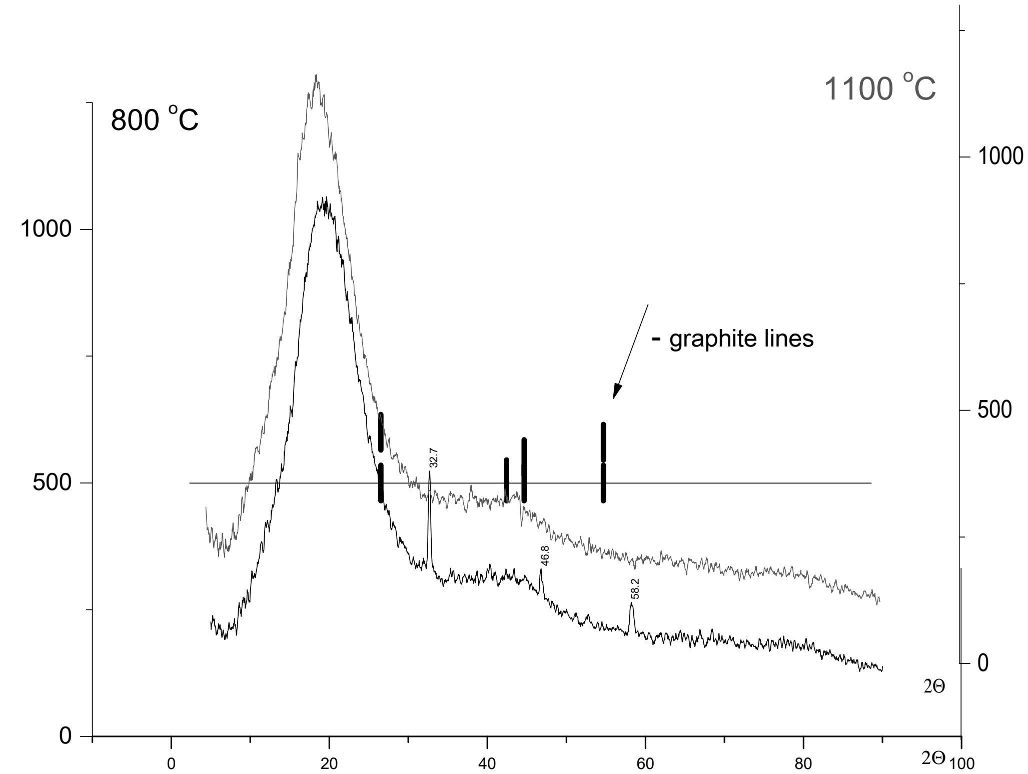

Figure S7. Powder X-ray diffraction patterns of **RF-800** (down) and **RF-1100** aerogels (up).

5. IR spectroscopy

The spectra of attenuated total reflection (ATR-FTIR) of **RF-1100** surface are presented at Figure S8. They contain three weak lines 2953.76, 2914.96 and 2848.84 cm^{-1}.

These lines are characteristic of graphene-like structures such as carbon nanotubes and reveal the appearance of "intergrain phase" at high-temperature pyrolysis. At a lower pyrolysis temperature (900 °C or less), no such lines are observed.

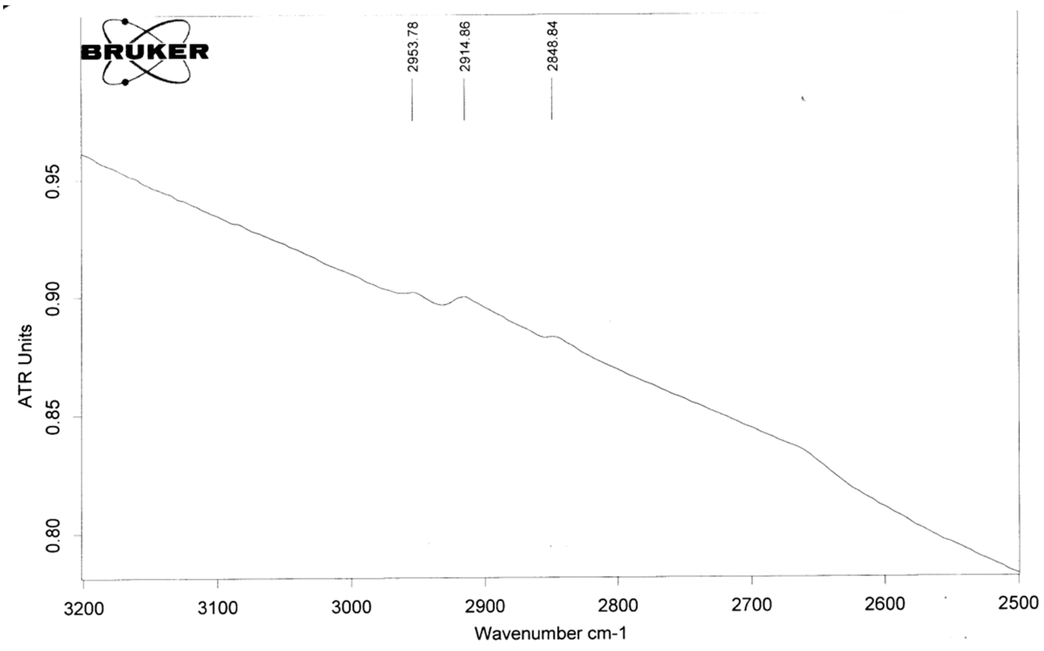

Figure S8. The ATR-FTIR spectrum of **RF-1100** aerogel surface.
